# A comprehensive analysis of the association of common variants of *ABCG2* with gout

**DOI:** 10.1038/s41598-017-10196-2

**Published:** 2017-08-30

**Authors:** Kuang-Hui Yu, Pi-Yueh Chang, Shih-Cheng Chang, Yah-Huei Wu-Chou, Li-An Wu, Ding-Pin Chen, Fu-Sung Lo, Jang-Jih Lu

**Affiliations:** 1 Division of Rheumatology, Allergy and Immunology, Chang Gung Memorial Hospital and Chang Gung University, Taoyuan, Taiwan; 2 Department of Laboratory Medicine, Chang-Gung Memorial Hospital at LinKou, Taoyuan, Taiwan; 3 Department of Medical Biotechnology and Laboratory Science, Chang Gung University, Tao-Yuan, Taiwan; 4 Department of Medical Research, Chang Gung Memorial Hospital and Chang Gung University, Taoyuan, Taiwan; 5 Division of Pediatric Endocrinology and Genetics, Chang Gung Memorial Hospital and Chang Gung University, Taoyuan, Taiwan

## Abstract

The objective of the present study was to determine whether there was an association between single nucleotide polymorphisms (SNPs) in *ABCG2* and gout. We recruited 333 participants including 210 patients with gout and 123 controls and genotyped 45 SNPs in both cohorts. We found that 24 SNPs in *ABCG2* are susceptibility loci associated with gout. Haplotype analysis revealed five blocks across the *ABCG2* locus were associated with an increased risk of gout with odds ratios (ORs) from 2.59–3.17 (all *P* < 0.0001). A novel finding in the present study was the identification of rs3114018 in block 3 and its association with increased gout risk. We found that the rs2231142T allele in block 2 and the rs3114018C-rs3109823T (C-T) risk haplotype in block 3 conferred the greatest evidence of association to gout risk (*P* = 1.19 × 10^−12^ and *P* = 9.20 × 10^−11^, respectively). Our study provides an improved understanding of *ABCG2* variations in patients with gout and, as shown by haplotype analysis, that *ABCG2* may have a role in gout susceptibility.

## Introduction

Gout is an arthritis that is characterized by elevated serum uric acid level, recurrent acute arthritis, and chronic tophaceous gout^1–3^. Epidemiological studies from several countries have found that the incidence and prevalence of gout may be increasing^1–5^. Moreover, women comprise approximately 5% of all patients with gout, but the incidence of gout in women has doubled in the past 20 years^3–5^. An increased serum uric acid concentration is because of either overproduction or under excretion of uric acid^1^. In over 90 percent of patients, gout is caused by the under excretion of uric acid^1^. Genome-wide association studies (GWAS) that scan the genome for common genetic variants associated with gout have greatly advanced our medical knowledge^2, 6^. The majority of genes associated with serum urate levels or gout are involved in the renal urate-transport system.

Gout is a complex disease with a multifactorial etiology involving genetic and environmental factors^4, 5^. Several molecules are associated with gout and hyperuricemia in various populations^7–20^. Moreover, several GWASs on gout and hyperuricemia have been performed to date, and more than 50 loci have been identified^2, 7, 9, 14, 18–20^. Recent GWAS have identified substantial associations between SNPs in *ABCG2* and uric acid concentration and gout in different ethnic groups^2, 7^. *ABCG2* (also known as *BCRP*) is located at a gout-susceptibility locus on chromosome 4q22, which was previously identified in several genome-wide linkage studies of gout^7, 12, 20^. ABCG2 mediates urate secretion in proximal renal tubule cells, the intestine, and the liver^2, 8, 9^. Furthermore, several studies have proposed that variations in *ABCG2* may be important in the etiology of gout^2, 7–17, 21^. To verify further the impact of polymorphisms in genes related to gout, we studied common genetic variability in *ABCG2* using a case-control study to clarify the association between SNPs or haplotypes at *ABCG2* with the risk of gout in a Chinese population.

## Methods

### Study population

All enrolled patients were recruited at the Chang Gung Memorial Hospital (CGMH) at Tao-Yuan County (Taiwan) from February 2013 to March 2016. The study was approved by the local ethics committee and Institutional Review Board of Chang Gung Memorial Hospital (IRB 101-4659A3, 101-2636A3, and CMRPG3C1421-3). All participants provided written informed consent documents before entering the study. The methods carried out in accordance with the approved study protocol. A diagnosis of gout was based on the 1977 American College of Rheumatology diagnostic criteria^22^. All blood specimens were sent to the clinical laboratory at our hospital, which is certified by the College of American Pathologists (CAP) from the United States. External quality control for laboratory data was assessed by participation in the CAP’s international survey proficiency testing program and the National Quality Control Program conducted by the Taiwanese government.

### SNP identification and genotyping

DNA from peripheral blood was isolated from 333 participants including 210 patients with gout and 123 individuals who are gout-free (controls). DNA were extracted from venous blood using standard procedures, including lysis of blood cells, protein hydrolysis using proteinase K, DNA purification by extraction with phenol-chloroform, and DNA precipitation with ethanol. Genomic DNA was isolated from lymphocytes of each participant using a QIAamp DNA Blood Mini Kit and the standard protocol of the manufacturer (Qiagen, Valencia, CA, USA) according to the manufacturer’s instructions. We followed strict quality control procedure. Forty-six SNPs were selected from a small scale preliminary study to identify gout-associated variants by targeted next-generation sequencing of ABCG2 gene^23^. Forty-six SNPs in *ABCG2* on chromosome 4q22 were genotyped in our 210 cases and 123 controls using the Sequenom Mass-ARRAY platform and the standard protocol recommended by the manufacturer (Sequenom, San Diego, CA, USA). The call rate was ≥99.4% for all SNPs. During quality control review of genotyping data, we excluded one SNP (rs386677040) from further analysis as it was out of Hardy–Weinberg equilibrium (HWE; *P* < 0.05) in controls. Ultimately, the 45 SNPs that were in HWE (*P* > 0.05) were tested in our study cohorts.

### Fine mapping of *ABCG2* and haplotype analysis

We calculated linkage disequilibrium (LD) coefficients and constructed haplotypes using Haploview version 4.2 (Mark Daly’s Laboratory, Massachusetts Institute of Technology/Harvard Broad Institute, Cambridge, MA, USA)^24^. For haplotype construction, genotype data from both case and control groups were used to estimate intermarker LD by measuring pairwise D′ and r^2^, and to define LD blocks^24, 25^.

### Statistical analysis

Categorical variables were expressed as percentages and were analyzed by chi-square (*χ* ^2^) test or Fisher’s exact test, as appropriate. Continuous variables were expressed as mean ± SD. All *P* values in this study were two sided, and *P* < 0.05 was considered statistically significant. SNP frequencies were tested for departure from HWE using an exact test in control subjects. Allele and genotype frequencies for each SNP were compared between patient and control cohorts using the *χ* ^2^ test. Odds ratios (ORs) and 95% confidence intervals (CIs) were calculated using logistic-regression analysis. In addition to obtaining nominal *P* values, empirical *P* values were generated by running 10,000 permutations using the Max (T) permutation procedure implemented in PLINK v1.07^26^. In addition, we applied Bonferroni correction and set the significance threshold for these analyses at α = 1.1 × 10^−3^, which corresponds to a stringent Bonferroni correction for testing 45 independent markers. All statistical analyses were performed using SPSS 20.0 (IBM Corp., Armonk, NY, USA). Each marker was tested for association using PLINK v1.07 (http://pngu.mgh.harvard.edu/purcell/plink/)^26^. Haploview (v4.2) was used for assessing LD patterns and haplotype association statistics^24^. Haplotype blocks were determined using the algorithm of Gabriel *et al*.^27^. An omnibus (or global) test of haplotype association was performed using PLINK. ORs and 95% CIs for haplotype-specific risks were calculated using VassarStats (http://vassarstats.net/).

## Results

### Characteristics of study subjects

Our study consisted of 333 participants of which 210 were patients with gout and 123 were controls. Detailed information of study participants is shown in Table 1. The mean age of affected individuals was 52.4 ± 12.9 years (range 20–85 years) with a male-to-female ratio of 201:9 (approximately 22.3:1), while the mean age of controls was 51.9 ± 11.83 years (range 27–81 years) with a male-to-female ratio of 107:16 (approximately 6.7:1) (Table 1). As gout primarily affects males, fewer females than males participated in this study. However, we found that there was no significant difference between cohorts in terms of age distribution (*P* = 0.588).

**Table 1:** Characteristics of the study participants enrolled in this study.

Study group	Patients	Control	*P* value
Number of participants	210	123	
Number of males (%) (male-to-female ratio)	95.7% (201:9)	87.0% (107:16)	0.004
Age (years) Age range (years)	52.6 ± 13.0 20–85	51.9 ± 11.9 27–81	0.588

### *ABCG2* SNP analysis

Forty-six SNPs were genotyped in patients (*n* = 210) and controls (*n* = 123). We calculated HWE for all SNPs and found that all were in HWE (*P* > 0.05) with the exception of rs386677040, which was excluded from further analysis. Detailed information of the 45 SNPs and the results of our association analysis with gout in the present study are presented in Table 2. Genomic position, nucleic acid composition, allele frequencies, summary OR, 95% CI, and significance level of these 45 SNPs are summarized in Table 2. Twenty-four SNPs (rs2231156, rs4148157, rs4693924, rs76979899, rs2725263, rs2054576, rs2622621, rs1481012, rs45499402, rs149027545, rs2231142, rs4148155, rs3114018, rs3109823, rs2725246, rs2725245, rs2622624, rs145778965, rs2725239, rs4148162, rs3841115, rs2622606, rs2622608, and rs2622609) were positively associated with gout risk. ORs of these 24 SNPs ranged from 2.32 to 3.29 (*P* values ranged from 1.70 × 10^−7^ to 1.82 × 10^−12^) (Table 2). The greatest evidence of association was found between the minor T allele of rs2231142 and an increased risk of gout, with a frequency of 0.586 in cases and 0.301 in controls (*P* = 1.19 × 10^−12^; Bonferroni corrected *P* = 5.36 × 10^−11^; OR = 3.29; 95% CI = 2.36–4.60). In contrast, 21 SNPs (rs1448784, rs4148160, rs2231164, rs34455506, rs2231148, rs12505410, rs200184409, rs5860118, rs397994425, rs45557042, rs11935697, rs3109824, rs2725256, rs2231138, rs3114017, rs2725254, rs12641369, rs4148152, rs2231137, rs1564481, and rs4148149) were associated with a decreased risk of gout (ORs ranged from 0.31 to 0.67, all *P* < 0.05). We found that rs3109824, rs2725256, rs3114017, rs2725254, and rs1564481 were not associated with gout in our affected cohort (*P* > 0.05). In addition, following adjustments for Bonferroni correction for testing 45 independent tests (α < 0.0011), we found that all SNPs with the exception of eight SNPs (rs45557042, rs11935697, rs3109824, rs2725256, rs2231138, rs3114017, rs2725254, and rs1564481) remained significantly associated with gout. We found that the inclusion of age and gender as covariates in logistic regression models did not substantially change the significance of the observed associations (data not shown).

**Table 2:** Characteristics of the polymorphisms in *ABCG2* and risk of gout.

SNP	Locus	Location	Reference/Variant	Allele frequency of controls	Allele frequency of patients	*P* value	OR	95% CI
rs1448784	3′-UTR	89012320	A/G	0.434	0.207	5.02 × 10^−10^	0.34	0.24–0.48
rs4148160	intron	89015090	C/T	0.344	0.158	3.28 × 10^−8^	0.36	0.25–0.52
rs2231164	intron	89015857	C/T	0.553	0.369	3.94 × 10^−6^	0.47	0.34–0.65
rs2231156	intron	89020427	C/A	0.234	0.440	9.30 × 10^−8^	2.58	1.81–3.68
rs4148157	intron	89020934	G/A	0.234	0.440	9.30 × 10^−8^	2.58	1.81–3.68
rs4693924	intron	89023224	G/A	0.234	0.440	9.30 × 10^−8^	2.58	1.81–3.68
rs34455506	intron	89024220	G/A	0.346	0.157	2.10 × 10^−8^	0.35	0.24–0.51
rs76979899	intron	89025241	C/T	0.232	0.443	4.96 × 10^−8^	2.63	1.85–3.75
rs2725263	intron	89026428	A/C	0.455	0.660	2.50 × 10^−7^	2.32	1.68–3.21
rs2231148	intron	89028478	T/A	0.337	0.158	8.58 × 10^−8^	0.37	0.25–0.54
rs2054576	intron	89028775	A/G	0.238	0.440	1.70 × 10^−7^	2.52	1.78–3.59
rs12505410	intron	89030841	T/G	0.393	0.167	7.70 × 10^−11^	0.31	0.21–0.44
rs2622621	intron	89030920	C/G	0.549	0.774	1.53 × 10^−9^	2.81	2.00–3.95
rs200184409	intron	89031978	T/A	0.398	0.179	5.38 × 10^−10^	0.33	0.23–0.47
rs5860118	intron	89032383	A/-	0.398	0.182	1.10 × 10^−9^	0.34	0.24–0.48
rs397994425	intron	89032388	A/-	0.398	0.182	8.88 × 10^−10^	0.34	0.24–0.48
rs1481012	intron	89039082	A/G	0.297	0.565	2.35 × 10^−11^	3.07	2.20–4.30
rs45557042	intron	89043462	G/A	0.118	0.052	2.15 × 10^−3^	0.41	0.23–0.74
rs45499402	intron	89043634	G/C	0.303	0.588	1.44 × 10^−12^	3.28	2.35–4.59
rs149027545	intron	89044180	G/C	0.301	0.584	1.82 × 10^−12^	3.26	2.33–4.56
rs11935697	intron	89044784	A/G	0.115	0.052	3.32 × 10^−3^	0.43	0.24–0.76
rs3109824	intron	89046935	T/A	0.248	0.200	1.48 × 10^−1^	0.76	0.52–1.10
rs2725256	intron	89050998	A/G	0.248	0.200	1.48 × 10^−1^	0.76	0.52–1.10
rs2231142	exon 5	89052323	G/T	0.301	0.586	1.19 × 10^−12^	3.29	2.36–4.60
rs2231138	intron	89053718	T/C	0.118	0.052	2.15 × 10^−3^	0.41	0.23–0.74
rs4148155	intron	89054667	A/G	0.303	0.586	2.12 × 10^−12^	3.25	2.33–4.55
rs3114017	intron	89055194	C/T	0.270	0.207	6.16 × 10^−2^	0.70	0.49–1.02
rs2725254	intron	89057664	C/T	0.246	0.202	1.91 × 10^−1^	0.78	0.53–1.13
rs12641369	intron	89059917	G/A	0.390	0.179	1.98 × 10^−9^	0.34	0.24–0.49
rs4148152	intron	89060909	T/C	0.385	0.176	2.31 × 10^−9^	0.34	0.24–0.49
rs2231137	exon 2	89061114	C/T	0.386	0.175	1.40 × 10^−9^	0.34	0.24–0.48
rs1564481	intron	89061265	C/T	0.246	0.202	1.91 × 10^−1^	0.78	0.53–1.13
rs4148149	intron	89062285	T/G	0.352	0.148	1.19 × 10^−9^	0.32	0.22–0.47
rs3114018	intron	89064581	A/C	0.585	0.818	6.12 × 10^−11^	3.19	2.24–4.55
rs3109823	intron	89064602	C/T	0.744	0.900	9.54 × 10^−8^	3.10	2.02–4.76
rs2725246	intron	89068498	G/A	0.631	0.829	1.12 × 10^−8^	2.82	1.96–4.06
rs2725245	intron	89068738	G/A	0.634	0.830	1.26 × 10^−8^	2.82	1.96–4.06
rs2622624	intron	89069406	T/C	0.634	0.831	1.03 × 10^−8^	2.84	1.97–4.08
rs145778965	intron	89075239	T/C	0.187	0.386	9.25 × 10^−8^	2.73	1.88–3.98
rs2725239	intron	89075623	C/A	0.620	0.831	1.26 × 10^−9^	3.01	2.10–4.34
rs4148162	intron	89080716	-/GTGA	0.623	0.836	6.94 × 10^−10^	3.08	2.14–4.44
rs3841115	intron	89080723	-/AGTG	0.626	0.838	6.29 × 10^−10^	3.09	2.14–4.46
rs2622606	intron	89084381	A/T	0.744	0.904	3.51 × 10^−8^	3.25	2.11–5.02
rs2622608	intron	89086744	A/T	0.622	0.831	1.52 × 10^−9^	2.99	2.08–4.29
rs2622609	intron	89088475	A/C	0.622	0.830	1.88 × 10^−9^	2.97	2.07–4.27

OR: odds ratio, CI: confidence interval.

### Linkage disequilibrium plot and haplotype analysis

**Figure 1:**
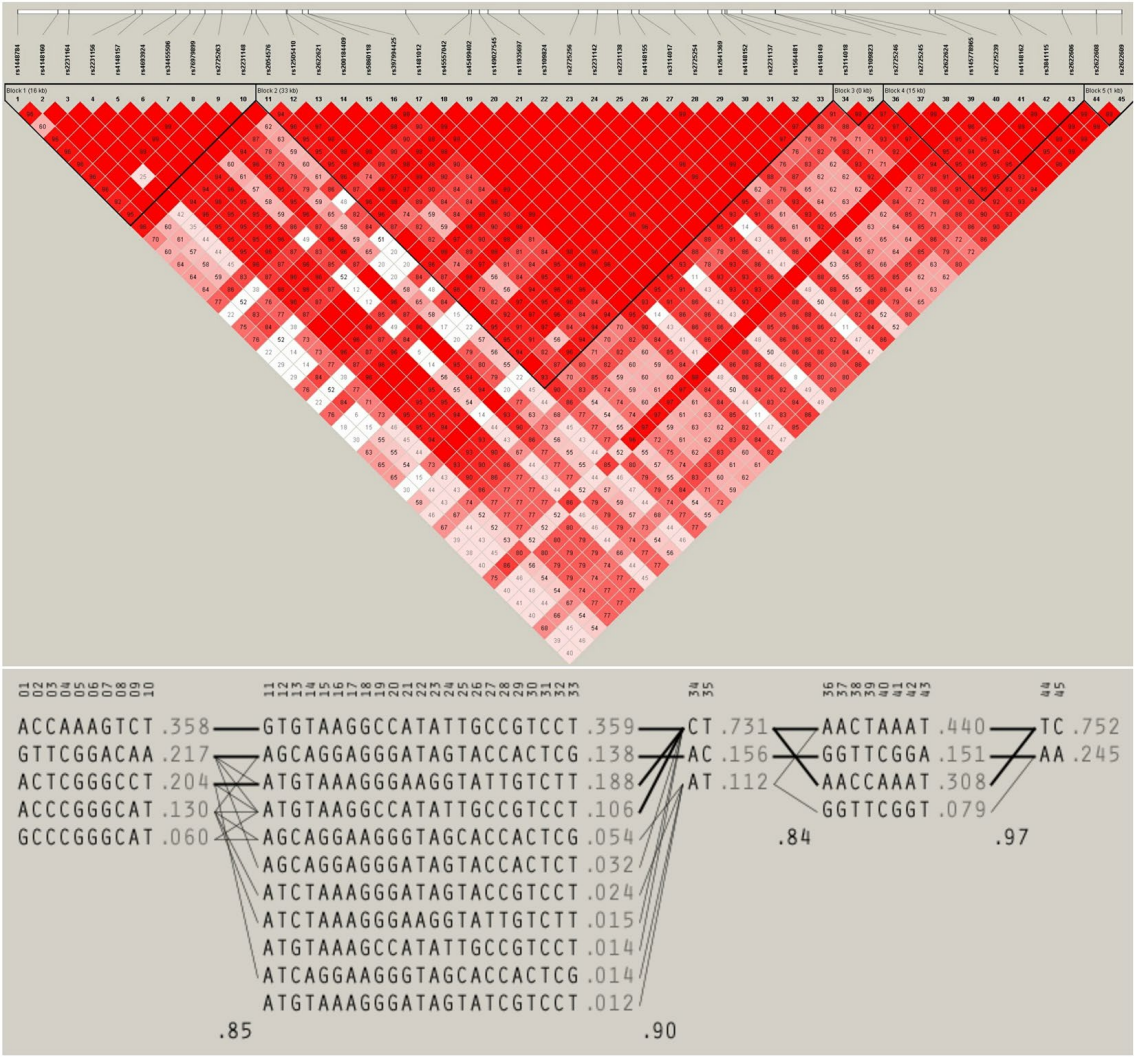
Linkage disequilibrium (LD) plots for *ABCG2* and haplotype block structure across the *ABCG2* locus. (**a**) Haploview plot defining haplotype block structure of the *ABCG2* locus. The white horizontal bar in the upper diagram illustrates the location of each SNP on a physical scale. Each box provides estimated statistics of the coefficient of determination (r^2^). The diamond without a number corresponds to a D′ of 1. (**b**) Haplotypes in the haplotype blocks spanning the *ABCG2* locus. There are five haplotype blocks across the region. Haplotype frequencies are shown to the right of each haplotype. SNP numbers across the top of the haplotypes correspond to those shown in the Haploview plot. A multiallelic D′ statistic, which indicates the level of recombination between two blocks, is shown in the crossing area. Connections from one block to the next are shown for haplotypes with a frequency >10% as thick lines and a frequency >1% as thin lines.

Using Haploview v4.2, we generated an LD plot of the 45 genotyped SNPs in *ABCG2* in our affected cohort (Fig. 1), and found that more than half of the SNPs tested in the present study were highly correlated with each other (r^2^ ≥ 0.80), of which 220 out of 990 (22.2%) pairs revealed perfect linkage disequilibrium (D′ = 1). Moreover, the haplotype block structure spanning *ABCG2* as derived by Haploview is shown in Fig. 1b with haplotype frequencies shown in both Fig. 1b and in Table 3. We found that there are five haplotype blocks across the *ABCG2* locus. Based on the model of Gabriel *et al*.^26^, we identified a total of 25 common haplotypes in these five blocks (Table 3), which span approximately 16 kb, 33 kb, <1 kb, 15 kb, and 1 kb derived from 5, 11, 3, 4, and 2 SNPs, respectively (Fig. 1a). Haploview predicted 34 possible connections of haplotypes for recombination between blocks at a frequency >1%. In addition, calculating the frequency of recombination between blocks as a value of a multiallelic D′ coefficient, we found values of 0.85 between blocks 1 and 2, 0.91 between blocks 2 and 3, 0.84 between blocks 3 and 4, and 0.97 between blocks 4 and 5 (Fig. 1b and Table 3). Among the identified 25 common haplotypes, five haplotypes were associated with an increased risk for gout (all permuted *P* < 0.0001) and seven haplotypes were associated with a decreased risk for gout (Table 3). The five haplotypes associated with an increased risk of gout following correction with 10,000 permutations were the A-C-C-A-A-A-G-T-C-T haplotype in block 1 (OR = 2.67; 95% CI = 1.87–3.81; *P* < 0.0001), the G-T-G-T-A-A-G-G-C-C-A-T-A-T-T-G-C-C-G-T-C-C-T haplotype in block 2 (OR = 2.59; 95% CI = 1.81–3.69; *P* < 0.0001), the C-T haplotype in block 3 (OR = 3.16; 95% CI = 2.21–4.50; *P* < 0.0001), the A-A-C-C-A-A-A-T haplotype in block 4 (OR = 2.76; 95% CI = 1.89–4.04; *P* < 0.0001), and the T-C haplotype in block 5 (OR = 2.94; 95% CI = 2.05–4.22; *P* < 0.0001) (Table 3). The haplotype that conferred the greatest risk was the rs3114018C-rs3109823T haplotype (C-T) in block 3, with a haplotype frequency of 0.816 in affected individuals and 0.585 in controls (*P* = 9.20 × 10^−11^; OR = 3.16; 95% CI = 2.21–4.50; Table 3).

**Table 3:** Haplotypes in the haplotype blocks spanning the *ABCG2* locus.

Gene*	Haplotype	Haplotype Frequency			OR	95% CI	Nominal P	Permuted P
		Sample	Gout	Control				
Block 1	ACCAAAGTCT	0.358	0.437	0.223	2.67	1.87–3.81	3.24 × 10^−8^	<0.0001
	GTTCGGACAA	0.217	0.152	0.327	0.37	0.25–0.54	1.25 × 10^−7^	<0.0001
	ACTCGGGCCT	0.204	0.21	0.194	1.11	0.75–1.65	0.617	1
	ACCCGGGCAT	0.130	0.136	0.119	1.16	0.72–1.88	0.532	1
	GCCCGGGCAT	0.060	0.047	0.081	0.56	0.30–1.06	0.071	0.6634
Block 2	GTGTAAGGCCATATTGCCGTCCT	0.359	0.436	0.23	2.59	1.81–3.69	9.40 × 10^−8^	<0.0001
	ATGTAAAGGGAAGGTATTGTCTT	0.188	0.172	0.218	0.74	0.50–1.11	0.144	0.955
	AGCAGGAGGGATAGTACCACTCG	0.138	0.09	0.222	0.35	0.22–0.55	2.16 × 10^−6^	<0.0001
	ATGTAAGGCCATATTGCCGTCCT	0.106	0.127	0.071	1.93	1.09–3.41	0.024	0.2925
	AGCAGGAAGGGTAGCACCACTCG	0.054	0.026	0.102	0.24	0.11–0.49	2.87 × 10^−5^	0.0002
	AGCAGGAGGGATAGTACCACTCT	0.032	0.029	0.037	0.77	0.32–1.85	0.575	1
	ATCTAAAGGGATAGTACCGTCCT	0.024	0.019	0.033	0.57	0.21–1.54	0.266	0.9993
	ATCTAAAGGGAAGGTATTGTCTT	0.015	0.019	0.007	2.35	0.49–11.14	0.196	0.9964
	ATGTAAAGCCATATTGCCGTCCT	0.014	0.019	0.004	4.71	0.59–37.89	0.105	0.7957
	ATCAGGAAGGGTAGCACCACTCG	0.014	0.017	0.008	2.05	0.42–9.93	0.364	0.9999
	ATGTAAAGGGATAGTATCGTCCT	0.012	0.007	0.021	0.34	0.08–1.45	0.128	0.9312
Block 3	CT	0.731	0.816	0.585	3.16	2.21–4.50	9.20 × 10^−11^	<0.0001
	AC	0.156	0.097	0.256	0.31	0.20–0.48	5.43 × 10^−8^	<0.0001
	AT	0.112	0.084	0.159	0.48	0.30–0.79	0.0031	0.0389
Block 4	AACTAAAT	0.44	0.442	0.435	1.03	0.75–1.42	0.854	1
	AACCAAAT	0.308	0.382	0.183	2.76	1.89–4.04	8.04 × 10^−8^	<0.0001
	GGTTCGGA	0.151	0.094	0.248	0.31	0.20–0.49	8.62 × 10^−8^	<0.0001
	GGTTCGGT	0.079	0.058	0.114	0.48	0.27–0.84	0.010	0.1335
Block 5	TC	0.752	0.829	0.622	2.94	2.05–4.22	2.51 × 10^−9^	<0.0001
	AA	0.245	0.167	0.378	0.33	0.23–0.47	9.14 × 10^−10^	<0.0001

OR: odds ratio, CI: confidence interval.

*SNPs are as numbered in Fig. 1a where block 1: SNPs 1-2-3-4-5-6-7-8-9-10; block 2: SNPs 11-12-13-14-15-16-17-18-19-20-21-22-23-24-25-26-27-28-29-30-31-32-33; block 3: SNPs 34-35; block 4: SNPs 36-37-38-39-40-41-42-43; and block 5: SNPs 44–45.

## Discussion

Gout is an increasing global health problem caused by multiple genetic and environmental factors. In recent years, many variants in a growing number of genes involved in the pathogenesis of gout and hyperuricemia have been identified^2, 9^. *ABCG2* is located at a gout-susceptibility locus on chromosome 4q22, which was previously identified in several genome-wide linkage studies of gout^7, 12, 20^. ABCG2, which is also known as breast cancer resistance protein (BCRP), is a high-capacity urate exporter, the dysfunction of which increases the risk of gout and hyperuricemia^8, 28^. ABCG2 mediates renal urate secretion as a urate efflux transporter in the brush-border membrane on the luminal surface of kidney proximal tubule cells^2, 8, 9^. In addition, ABCG2 is expressed at high levels in the intestine and liver^27^ and functions as an efflux transporter for many drugs and molecule substrates, including anticancer agents, antibiotics, antivirals, HMG-CoA reductase inhibitors, flavonoids, allopurinol, and uric acid^28–34^.

Using haplotype analyses, we found five blocks of LD that were significantly associated with gout. Moreover, an LD plot of *ABCG2* demonstrated extensive correlation among 45 SNPs. Based on measures of r^2^, perfect linkage (r^2^ = 1) was detected in 220 out of the 990 pairs (D′ = 1) and strong LD (1 > |D′| ≥ 0.8) was detected in more than half of variant pairs. A novel finding in the present study was the identification of rs3114018 in block 3 and its association with increased gout risk. In addition, the minor T allele of rs2231142 in the second block of *ABCG2* was associated with an increased risk of gout (OR = 3.29; 95% CI = 2.36–4.60), a finding that was similarly reported in previous studies in other populations^7, 10–12, 17, 21, 33, 35–37^.

Two independent functional studies of *ABCG2* found that the Q141K (rs2231142) polymorphism occurs in a highly conserved region of the gene and is a loss-of-function mutation^8, 33^. These studies found that the rs2231142 risk allele resulted in a urate secretory transporter with a 53% reduced ability to transport urate in *Xenopus* oocytes^8^ and HEK293 membrane vesicles^33^. Moreover, *Abcg2*-knockout mice had increased serum uric acid levels and renal urate excretion, and decreased intestinal urate excretion^28^. Furthermore, Woodward *et al*.^37^ demonstrated the utility of using small molecules to correct the Q141K defect in expression and function as a potential therapeutic approach for hyperuricemia and gout. The association between the rs2231142T allele and the risk of gout has been replicated in many diverse study populations including Caucasian^7, 11, 12, 33^, African^7^, Japanese^20, 33, 35^, Mexican-American^12^, Native American^12^, Han Chinese^10, 17, 36^, and New Zealand Pacific Island ancestry^11^. These findings indicate that *ABCG2* may have specific and important functions in the pathology of gout. However, an association between rs2231142 and gout has not been found in Maori populations^11^ and some studies in the Chinese population^21, 38^. The reason for this discrepancy is not known, but the difference may be because of either differences in gene structure or sampling bias^13^. Furthermore, an additional confounding factor is that the etiology of gout is linked to various genetic and environmental factors such as lifestyle and diet^1, 5, 35, 39^. However, the baseline socioeconomic status and diet habit were not available in the database, so we were unable to perform the analysis.

In this study, we thoroughly captured common genetic variation across *ABCB2* and performed a comprehensive evaluation of common SNPs at *ABCB2* associated with gout risk. Using haplotype analysis, we found five haplotype blocks that were associated with an increased risk of gout: block 1 with an OR of 2.67 (95% CI = 1.87–3.81), block 2 with an OR of 2.59 (95% CI = 1.81–3.69), block 3 with an OR of 3.16 (95% CI = 2.21–4.50), block 4 with an OR of 2.76 (95% CI = 1.89–4.04), and block 5 with an OR of 2.94 (95% CI = 2.05–4.22; all *P* < 0.0001) (Table 3). Our results, combined with those from previous studies, suggest that genetic variation in *ABCG2* may influence gout susceptibility in the Han Chinese population. Consistent with the genetic susceptibility identified in patients with gout in several other populations, we observed that the minor allele of rs2231142 was associated with an increased risk for gout^7, 10–12, 17, 20, 33, 35–37^, while we found other SNPs in the present study that may confer a protective effect on susceptibility to gout. This finding is consistent with the hypothesis of two functional polymorphisms near the SNPs evaluated in this study, one that increases the risk of developing gout whereas the second confers a protective effect^40^. In addition, considering that the genomic regions of the five SNP haplotype blocks are characterized by high LD, we postulate that such SNPs are likely to tag any hitherto unidentified common SNPs in the candidate gene. For example, two recent studies from northwest China^21, 38^ found a significant difference in mean serum urate levels between a novel SNP, rs3114018, in *ABCG2* and gout risk, which is consistent with the findings of the present study. In addition to rs2231142 in block 2, the greatest evidence of association in the present study was between the C-T risk haplotype of rs3114018 and rs3109823 in block 3. To the best of our knowledge, the relationships demonstrated in the present study between SNPs in blocks 1, 3, 4, and 5 with gout, such as the novel SNP rs3114018 in block 3 have not been previously observed until recently^21, 38^. Of note, rs2231137 is located in the same block with rs2231142 (block 2), resulting in a V12M substitution (p = 1.4 × 10^−9^). Our findings and previous studies^10, 17^ indicated that V12M substitution was associated with a decreased risk of hyperuricemia and/or gout. However, *in vitro* functional assays showed that V12M substitution did not result in any changes in protein expression and risk to phenotypes such as serum urate levels and gout^33, 41^. Further studies are required to elucidate the functional contributions of these novel SNPs in these genomic regions or blocks that confer increased risk for gout.

The present study had the following limitations. First, although we could identify genetic associations with gout, we could not elucidate the underlying causal mechanisms. Nonetheless, our findings with rs2231142 and rs3114018 are consistent with those of studies of other populations, which highlight their robustness and support for a role in gout. Second, considering the marked difference in SNP minor allele frequencies among populations, ethnic differences may exist, which would confound the identification of genetic risk factors for gout^2, 9, 42^. Future studies should incorporate larger sample sizes to verify present findings across more populations. Finally, the biological functions of other SNPs in *ABCG2* have not been fully characterized, and therefore, the findings from the present study require functional confirmation by future expression studies.

In conclusion, this large-scale thorough evaluation of SNPs has identified common genetic variants in *ABCG2* that are associated with gout risk. None of the tested SNPs, with the exception of rs2231142, which were identified as significant in this study were listed among the most significant results of three recently conducted GWAS on gout^7, 12, 20^. In addition to rs2231142, haplotype analysis of polymorphisms in *ABCG2* revealed SNP-derived haplotypes associated with gout risk. Further identification of the functional and causal variant(s) in *ABCG2* will lead to a better understanding of the mechanism underlying the development of gout pathologies.
